# Skeletal Muscle Loss and Octogenarian Status Are Associated with S-1 Adjuvant Therapy Discontinuation and Poor Prognosis after Pancreatectomy

**DOI:** 10.3390/cancers13164105

**Published:** 2021-08-15

**Authors:** Mariko Tsukagoshi, Norifumi Harimoto, Kenichiro Araki, Norio Kubo, Akira Watanabe, Takamichi Igarashi, Norihiro Ishii, Takahiro Yamanaka, Kei Hagiwara, Kouki Hoshino, Ryo Muranushi, Toshiki Yajima, Ken Shirabe

**Affiliations:** 1 Department of Innovative Cancer Immunotherapy, Gunma University Graduate School of Medicine, 3-39-22 Showa-Machi, Maebashi 371-8511, Japan; marikot@gunma-u.ac.jp (M.T.); yajimato@gunma-u.ac.jp (T.Y.); 2 Division of Hepatobiliary and Pancreatic Surgery, Integrative Center of General Surgery, Gunma University Hospital, 3-39-15 Showa-Machi, Maebashi 371-8511, Japan; karaki@gunma-u.ac.jp (K.A.); nkubo@gunma-u.ac.jp (N.K.); akira_watanabe@gunma-u.ac.jp (A.W.); takamichi.iga@gunma-u.ac.jp (T.I.); n.ishii@gunma-u.ac.jp (N.I.); tyamanaka@gunma-u.ac.jp (T.Y.); kei-hagiwara@gunma-u.ac.jp (K.H.); h.kouki.915@gunma-u.ac.jp (K.H.); m1820020@gunma-u.ac.jp (R.M.); kshirabe@gunma-u.ac.jp (K.S.)

**Keywords:** adjuvant chemotherapy, elderly patients, outcome, pancreatic cancer, sarcopenia

## Abstract

**Simple Summary:**

Significant advances in surgical techniques and perioperative care, together with adjuvant chemotherapy, have contributed to the increasing number of patients with pancreatic cancer undergoing surgery. However, the results of some studies suggest that the postoperative complications and mortality might be higher in elderly patients undergoing pancreatectomy. We aimed to identify the utility of S-1 adjuvant chemotherapy in elderly patients with resected pancreatic cancer. In our cohort of 80 patients, including 16 octogenarians, univariate and multivariate analyses revealed that S-1 adjuvant chemotherapy was associated with improved prognosis in patients with pancreatic cancer. However, we also observed that skeletal muscle loss and age of 80 years or older predicted the failure to complete adjuvant chemotherapy with S-1. We propose that evaluation of skeletal muscle mass should be considered as a useful preoperative assessment approach for determining feasibility of adjuvant chemotherapy in elderly patients.

**Abstract:**

The efficacy and prognosis of adjuvant chemotherapy for resected pancreatic cancer remain unclear. We investigated the utility and risk factors of S-1 adjuvant chemotherapy in patients with pancreatic cancer undergoing pancreatectomy. This study comprised 80 patients, including 58 patients who received S-1 adjuvant chemotherapy. Skeletal muscle loss was defined using cutoff values of skeletal muscle mass index. In total, 16 (20%) octogenarian patients underwent pancreatectomy. Skeletal muscle loss was present in 56 (70%) patients. The entire course of S-1 adjuvant chemotherapy for 6 months was completed in 33 patients (41%). S-1 adjuvant chemotherapy <6 months was an independent prognostic indicator of poor overall survival. Patients who completed S-1 adjuvant chemotherapy exhibited significantly longer overall and relapse-free survival rates than those did not complete the chemotherapy (*p* < 0.0001 and *p* = 0.0003, respectively). Being an octogenarian and skeletal muscle loss were independent variables associated with the discontinuation of S-1 adjuvant chemotherapy. Finally, the S-1 adjuvant chemotherapy rates were 6.3% (1/16) and 28.6% (16/56) in octogenarian patients and those with skeletal muscle loss, respectively. S-1 adjuvant chemotherapy completion was associated with improved prognosis in patients with pancreatic cancer. Skeletal muscle loss and octogenarian status predicted the failure of S-1 adjuvant chemotherapy completion.

## 1. Introduction

Pancreatic cancer, one of the most lethal malignancies, is associated with poor prognosis, with a five-year relative survival rate of only 9% [1], and surgical resection is the only potentially curative treatment option. The safety profile of surgical resection of pancreatic cancer has improved with advances in surgical techniques and perioperative care during the last decade, and postoperative outcomes have displayed continuous improvement. Therefore, an increasing number of patients, including those who are elderly, are considered for surgical resection.

The continual increase in the aging population worldwide is accompanied with an increase in the number of elderly patients eligible for surgical procedures. In Japan, the percentage of individuals aged 65 years and older, which was 28.4% in 2019, is projected to exceed 35% in 2040 [2]. Additionally, the proportion of individuals aged 80 years and older, i.e., octogenarians, was 8.9% in 2019 in Japan. Several studies have previously reported that pancreatic resection can be performed safely in certain elderly patients [3,4,5,6]. However, other reports have indicated increased rates of postoperative complications and mortality and longer hospital stays in elderly patients undergoing resection for pancreatic malignancies [7,8,9], and the survival benefit of surgical resection in elderly patients remains uncertain [10].

Adjuvant chemotherapy is a standard option in patients undergoing pancreatic cancer resection. Specifically, the Japan Adjuvant Study Group of Pancreatic Cancer (JASPAC)-01 trial demonstrated that S-1 was an effective adjuvant chemotherapy in Japanese patients [11]. The efficacy of chemotherapy for the prognosis of elderly patients with pancreatic cancer is unclear because few studies have investigated the feasibility of adjuvant chemotherapy in these patients.

Numerous recent studies have suggested an association between sarcopenia and postoperative outcomes in patients with pancreatic cancer undergoing pancreatectomy [12]. The impact of sarcopenia, which is defined as loss of skeletal muscle mass and strength [13], has also been investigated in patients with advanced pancreatic cancer during chemotherapy. For example, sarcopenia was an independent prognostic factor in patients with pancreatic cancer receiving FOLFIRINOX (leucovorin, 5-fluorouracil, irinotecan, and oxaliplatin) [14]. However, the impact of preoperative skeletal muscle mass on outcomes in patients undergoing adjuvant chemotherapy is unclear.

The purpose of the present study was to investigate the utility of S-1 adjuvant chemotherapy and risk factors associated with worse outcomes in patients with pancreatic cancer undergoing pancreatectomy.

## 2. Materials and Methods

### 2.1. Patient Selection

This was a retrospective analysis of 80 patients with pancreatic cancer who underwent pancreatic resection between January 2016 and August 2019 in the Department of Hepatobiliary and Pancreatic Surgery at Gunma University Hospital. The study was approved by the Ethics Committee of the study hospital (No. 1378), and all clinical samples were used in accordance with institutional guidelines and the Declaration of Helsinki after signed informed consent was obtained from all participants.

### 2.2. Treatment and Data Collection

Baseline clinical and demographic characteristics and treatment-related details of all patients were collected from the medical records. Surgical procedures were performed according to institutional policies and the institutional cancer board recommendations. Eight patients with borderline resectable pancreatic cancer who received neoadjuvant chemotherapy with gemcitabine plus nab-paclitaxel regimen were included. The Clavien–Dindo classification was used to evaluate postoperative complications [15]. Complications requiring surgical intervention during the first 30-day period after surgery were defined as Clavien–Dindo grade III or higher complications. Resected tumors were classified according to the TNM staging system of the International Union Against Cancer [16].

The patients who received S-1 adjuvant chemotherapy were treated following a 6-week schedule, with each course comprising S-1 doses of 80, 100, and 120 mg/day for 4 weeks based on body surface areas of <1.25 m^2^, between ≥1.25 m^2^ and <1.5 m^2^, and ≥1.5 m^2^, respectively, followed by 2 weeks of rest. Progression-free survival was defined as the period from the date of surgery until the date of documented disease progression or death from any cause. Overall survival (OS) was defined as the period from the date of surgery to the date of death from any cause.

### 2.3. Definition and Determination of Skeletal Muscle Loss

In all patients, the area of skeletal muscle mass at the inferior aspect of the third lumbar vertebra (L3) was measured using computed tomography images obtained within 30 days prior to surgery. To minimize measurement bias, a trained investigator blinded to all anthropometric and surgical characteristics identified and measured the skeletal muscle area using a dedicated processing system, the volume analyzer SYNAPSE VINCENT (Fujifilm Medical, Tokyo, Japan). Cross-sectional area (cm^2^) of the skeletal muscle in the L3 region were measured by rough manual outlining on computed tomography images, and total cross-sectional area of the segmented tissue was automatically calculated. Muscle area computed from each image was normalized as follows: skeletal muscle index = cross-sectional area of the skeletal muscle in the L3 region/height [2] (cm^2^/m^2^). The cutoff values for skeletal muscle mass index were defined as 42 cm^2^/m^2^ for male patients and 38 cm^2^/m^2^ for female patients, according to the Japan Society of Hepatology guidelines [17]. Skeletal muscle loss was defined based on these cutoff values, and the patients were classified accordingly into those with and without skeletal muscle loss.

### 2.4. Evaluation of Inflammatory and Nutritional Factors

We evaluated inflammatory and nutritional status of patients using prognostic nutritional index and modified Glasgow prognostic score (mGPS) [18], which were calculated based on the data at the time of first visit. Preoperative prognostic nutritional index was calculated as 10× serum albumin (g/dL) + 0.005 × total lymphocyte count (/mm^3^) [19]. The mGPS was calculated based on C-reactive protein (CRP) and albumin concentrations as follows. Patients with an elevated CRP level and hypoalbuminemia (<3.5 g/dL) were allocated a score of 2, patients with only an elevated CRP level (>1.0 mg/dL) were allocated a score of 1, and patients with normal CRP (≤1.0 mg/dL) and albumin (≥3.5 g/dL) levels were allocated a score of 0 [20].

### 2.5. Statistical Analysis

Categorical variables were assessed using the chi-square test or Fisher’s exact test, as appropriate. The Mann–Whitney *U* test was used to analyze continuous variables. Survival curves were estimated using the Kaplan–Meier method, and the log-rank test was used to analyze differences between the curves. Survival time was calculated from the date of surgery. Univariate and multivariate analyses of prognostic factors were calculated with the Cox proportional hazards model. The impact of factors on completion of S-1 adjuvant chemotherapy was examined using univariate and multivariate logistic regression analyses. A *p*-value of <0.05 was considered to indicate statistical significance. All statistical analyses were performed using the JMP Pro 14 statistical software (SAS Institute, Cary, NC, USA).

## 3. Results

### 3.1. Clinical Characteristics

The clinical characteristics of the patients are summarized in Table 1. There were 43 male and 37 female patients, with a median age of 72 (range, 42–88) years at the time of surgery. The study cohort included 16 (20%) octogenarian patients who underwent pancreatectomy, and 56 (70%) patients had skeletal muscle loss according to the skeletal muscle mass index. Surgical procedures included pancreaticoduodenectomy (*n* = 52), distal pancreatectomy (*n* = 26), and total pancreatectomy (*n* = 2). The R0 resection rate was 79%. There were 19 (24%) Clavien–Dindo grade ≥III complications. In total, 33 (41%) patients completed the 6-month adjuvant chemotherapy with S-1, whereas S-1 adjuvant chemotherapy was not administered in 22 patients due to poor general condition (*n* = 14), no consent (*n* = 4), early postoperative recurrence (*n* = 2), other concurrent cancer (*n* = 1), and nab-paclitaxel plus gemcitabine as adjuvant setting (*n* = 1). The planned S-1 adjuvant chemotherapy was discontinued in 25 patients because of chemotherapy-related adverse events (*n* = 11) and postoperative recurrence (*n* = 14). Recurrence was observed in 70% of the patients. Disease recurrence during S-1 adjuvant chemotherapy was observed in 16 patients.

### 3.2. Prognostic Factors Associated with OS

Univariate and multivariate analysis were performed to analyze factors associated with OS in patients with pancreatic cancer who underwent pancreatectomy (Table 2). Univariate analysis revealed that octogenarian status (*p* = 0.008), mGPS ≥1 (*p* = 0.029), and S-1 adjuvant chemotherapy for <6 months (*p* < 0.0001) were significantly associated with OS. Multivariate analysis revealed that S-1 adjuvant therapy for <6 months (odds ratio [OR], 3.99; 95% confidence interval (CI), 1.76–9.03; *p* = 0.0009) were independent prognostic indicators of poor survival.

### 3.3. Association between S-1 Adjuvant Chemotherapy Completion and Prognosis

**Figure 1:**
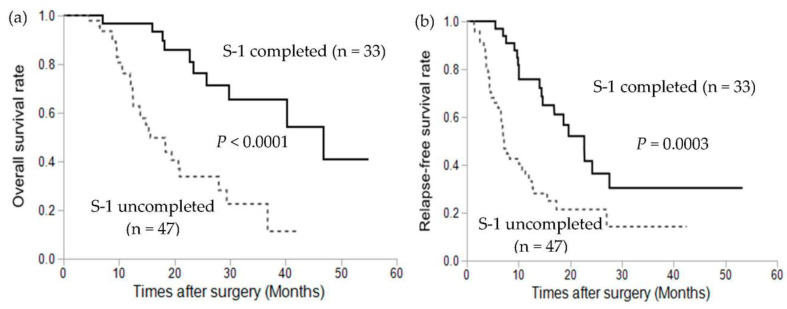

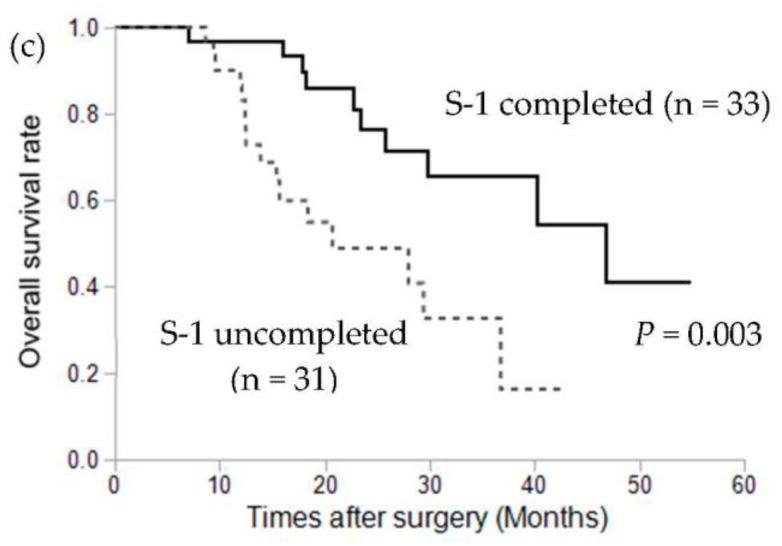
Kaplan–Meier plots of (**a**) overall survival, (**b**) relapse-free survival, and (**c**) overall survival of 64 patients, with the exclusion of those with recurrence during S-1 adjuvant chemotherapy.

The prognostic significance of S-1 adjuvant chemotherapy is shown in Figure 1. The median follow-up period was 18.2 months (range, 4.5 to 54.9 months) for all patients. The median OS was significantly longer in patients who completed S-1 adjuvant chemotherapy compared to those who did not complete the treatment (41.4 vs. 15.5 months, *p* < 0.0001; Figure 1a). The median relapse-free survival was significantly longer in patients who completed S-1 adjuvant chemotherapy compared to those who did not complete S-1 adjuvant chemotherapy (21.8 vs. 7.0 months, *p* = 0.0003; Figure 1b).

Among the 47 patients who did not complete S-1 adjuvant chemotherapy, 16 patients experienced recurrence during S-1 adjuvant chemotherapy. We analyzed the prognostic significance of S-1 adjuvant chemotherapy in 64 patients after the exclusion of these 16 patients and found that the median OS was significantly longer in patients who completed S-1 adjuvant chemotherapy compared with those who did not complete the S-1 adjuvant chemotherapy (41.4 vs. 19.4 months, *p* = 0.003; Figure 1c).

### 3.4. Factors Associated with S-1 Adjuvant Chemotherapy Completion

**Figure 2:**
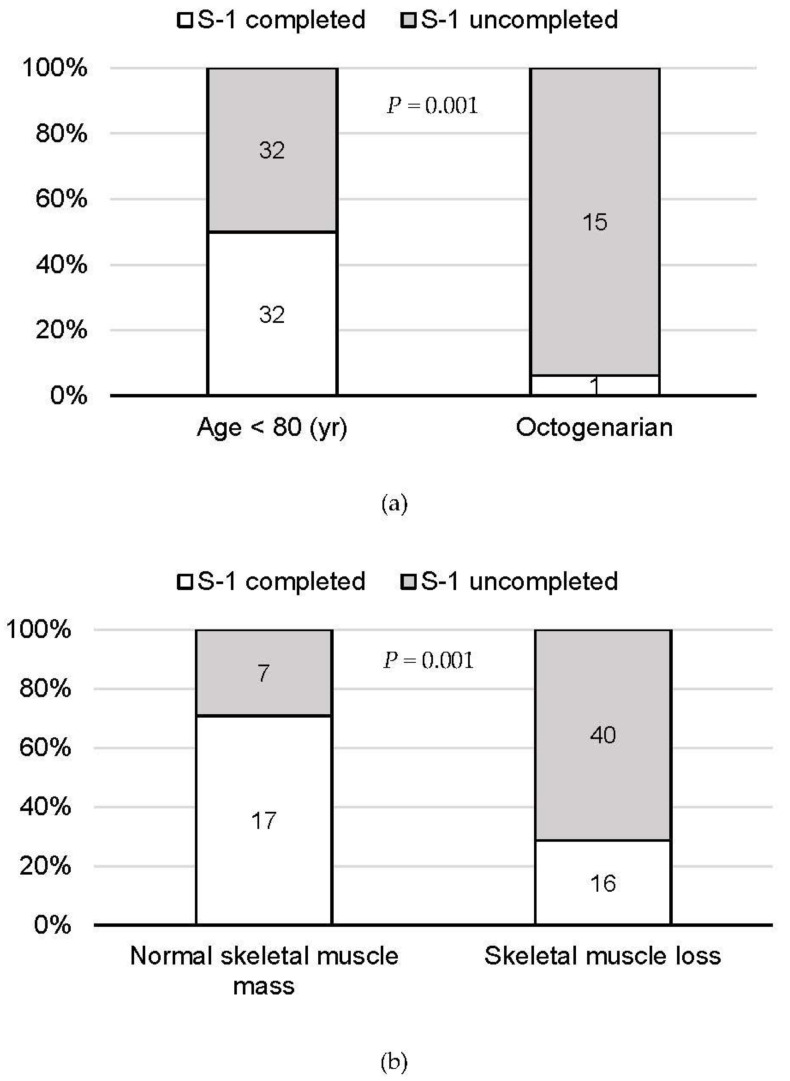
S-1 adjuvant therapy completion rates in (**a**) octogenarian patients and (**b**) those with skeletal muscle loss.

Based on our analysis showing that the completion of S-1 adjuvant chemotherapy was significantly associated with prognosis after surgery in patients with pancreatic cancer, we next investigated risk factors associated with S-1 adjuvant chemotherapy. By univariate and multivariate analyses, octogenarian status and skeletal muscle loss were independent factors associated with S-1 adjuvant chemotherapy discontinuation (OR, 15.94; 95%CI, 1.82–140.06; *p* = 0.013 and OR, 6.37; 95%CI, 1.99–20.37; *p* = 0.002, respectively; Table 3). The rate of S-1 adjuvant chemotherapy completion was 6.3% (1/16; *p* = 0.001; Figure 2a) among octogenarian patients and 28.6% (16/56; *p* = 0.001; Figure 2b) among patients with skeletal muscle loss.

## 4. Discussion

In the present study, which aimed to determine factors that might affect adjuvant chemotherapy completion and prognosis after pancreatectomy in patients with pancreatic cancer, our analyses revealed that failure to complete S-1 adjuvant chemotherapy was an independent prognostic indicator of poor survival. Moreover, octogenarian status and skeletal muscle loss were independent variables associated with the discontinuation of S-1 adjuvant chemotherapy. Overall, these findings suggest that surgical indications should be evaluated with more care and stronger perioperative management should be provided for octogenarians and patients with skeletal muscle loss.

Since the JASPAC-01 trial [11], S-1 has been recognized as a standard adjuvant chemotherapy and is routinely recommended for patients with resected pancreatic cancer in Japan. Both OS and relapse-free survival of patients with resected pancreatic cancer are significantly higher with S-1 adjuvant chemotherapy than with gemcitabine (median OS, 46.5 vs. 25.5 months; median relapse-free survival, 22.9 vs. 11.3 months) [11]. In the present study, S-1 adjuvant chemotherapy completion was an independent prognostic factor and patients who completed S-1 adjuvant chemotherapy experienced significantly longer overall and relapse-free survival compared to those who did not complete the treatment (median OS, 41.4 vs. 15.5 months; median relapse-free survival, 21.8 vs. 7.0 months).

In parallel, with the increasing percentage of elderly population, surgeons are increasingly faced with the prospect of performing surgery and administering adjuvant chemotherapy in elderly patients. In the present study, the median age was 72 years, with 16 (20%) patients aged over 80 years. Numerous studies have investigated the outcomes of pancreatic surgery in elderly patients including octogenarian patients [21]. Hardacre et al. reported that pancreatectomy could be performed in patients over the age of 80 years with low risk of mortality but that the procedure was associated with significant morbidity [22]. Hatzaras et al. reported that there were no significant differences in perioperative complication rate and OS between the patients aged younger than 80 years and those older than 80 years [23]. The overall perioperative complication and postoperative mortality rates in octogenarian patients appear to be similar to those in younger patients [24,25]. However, few studies have investigated outcomes of postoperative adjuvant chemotherapy in elderly patients with pancreatic cancer. Patients 70 years and older are less likely to receive adjuvant chemotherapy although this treatment approach is associated with prolonged survival [26]. Aoyama et al. reported that the safety and feasibility of S-1 adjuvant chemotherapy were similar between patients older than 70 years of age and those younger than 70 years of age [27]. In the JASPAC-01 trial [11], including participants aged 20 years or older, the median age of patients assigned to the S-1 arm was 66 (range, 60–73) years. Most clinical trials exclude patients aged over 80 years. Therefore, the safety and feasibility of adjuvant chemotherapy for octogenarian patients with pancreatic cancer remains controversial.

The purpose of adjuvant chemotherapy is to eradicate micrometastases and to prevent early tumor recurrence. S-1 adjuvant chemotherapy requires 6 months of treatment for patients with resected pancreatic cancer. In the JASPAC-01 trial [11], 52 of the 187 patients (28%) who received S-1 adjuvant chemotherapy discontinued treatment before completion. The most common reasons for discontinuation were adverse events in 40 (21%) patients and recurrence in 9 (5%) patients. Shimoda et al. reported that the planned S-1 adjuvant chemotherapy was completed in 11 (38%) of 29 patients [28]. In the present study, 33 (41%) patients completed S-1 adjuvant chemotherapy whereas 22 (28%) patients were untreated and 25 (31%) patients discontinued S-1 adjuvant chemotherapy before completion. We demonstrated that octogenarian status and skeletal muscle loss were independent variables associated with the failure to complete S-1 adjuvant chemotherapy.

In general, elderly patients often have comorbidities and age-related issues including a progressive decrease in muscle mass and strength. Loss of muscle mass is termed sarcopenia [29]. The prevalence of sarcopenia ranges from 30–65% in studies on patients with pancreatic cancer [30,31]. Sarcopenia is a significant predictor of poor prognosis in patients with unresectable pancreatic cancer receiving systemic chemotherapy [14,32]. A recent study has described the association of sarcopenia with postoperative complications after pancreatectomy. Centonze et al. reported that sarcopenia was an independent risk factor for postoperative complications and was related to a higher rate of grade C postoperative pancreatic fistula following pancreatoduodenectomy [33]. However, no study has reported the significance of sarcopenia in patients with resected pancreatic cancer receiving adjuvant chemotherapy. Our analyses revealed that the rate of S-1 adjuvant chemotherapy completion was significantly lower in patients with skeletal muscle loss than in those without skeletal muscle loss. Sarcopenia was also shown to be associated with toxicity caused by 5-fluorouracil-based chemotherapy [34]. Cousin et al. found that low skeletal muscle mass was associated with severe toxicity events in patients included in phase I trials [35]. Several candidate mechanisms have been proposed to underlie the increased risk of toxicity associated with sarcopenia. Sarcopenia might be related to possible drug overdose because chemotherapeutic drug dosages are conventionally determined based on body surface area [35,36]. Inflammation has been suggested as another mechanism since sarcopenia is often associated with inflammation. However, the relationship between sarcopenia and the risk of toxicity is not well established.

Kinoshita et al. compared octogenarian patients with pancreatic cancer who underwent pancreatectomy with those who received chemotherapy as an initial treatment and found that pancreatectomy was safe and feasible, although the prognosis was not superior to chemotherapy [37]. In the present study, there was an increased risk of failure to complete adjuvant chemotherapy in octogenarian patients and in those with skeletal muscle loss, which limited the overall benefit of pancreatectomy. Careful patient selection and perioperative management are therefore important in pancreatectomy.

The present study has several limitations. First, this was a retrospective observational study with a small sample size, particularly comprising octogenarians, and larger prospective studies are warranted to confirm and update the study conclusions. Second, there was a selection bias in the study; pancreatectomy is often avoided in elderly patients because of high morbidity and mortality rate. Third, the indications for adjuvant chemotherapy and initiation dose were based primarily on the attending physician, which could have led to the selection bias for patient inclusion for adjuvant chemotherapy. Fourth, neoadjuvant chemotherapy was not analyzed in this study. In fact, neoadjuvant chemotherapy is an increasingly adopted strategy for patients with resectable pancreatic cancer, and further studies are necessary to confirm and update the conclusions obtained in this study. Despite these limitations, the present study demonstrated that skeletal muscle loss and octogenarian status were predictors of the discontinuation of adjuvant chemotherapy with S-1 after pancreatectomy in patients with pancreatic cancer.

## 5. Conclusions

Octogenarian status and skeletal muscle loss were independent variables associated with the completion of S-1 adjuvant chemotherapy in patients with pancreatic cancer undergoing pancreatectomy. Therefore, patients younger than 80 years of age without sarcopenia should be considered as good candidates for surgical resection. However, the increasing number of elderly patients with cancer is undeniable. Evaluation of skeletal muscle mass is a good preoperative assessment tool for determining feasibility of adjuvant chemotherapy. In future, active perioperative nutrition and rehabilitation should be considered for the prevention of sarcopenia and successful completion of adjuvant chemotherapy, especially in elderly patients with pancreatic cancer.

## Figures and Tables

**Table 1:** Patient characteristics.

Factors	Patients (N = 80)
Age, years	72 (42–88)
Octogenarian	16 (20%)
Sex (male/female)	43/37
Skeletal muscle loss	56 (70%)
ASA PS classification (I/II/III/IV/V/VI)	1/67/12/0/0/0
Operation	
pancreaticoduodenectomy	52
distal pancreatectomy	26
total pancreatectomy	2
Disease stage	
IA, IB, IIA	28
IIB, IV	52
R0 resection	63 (79%)
Complications (Clavien–Dindo grade ≥III)	19 (24%)
S-1 adjuvant therapy completion	33 (41%)
Recurrence	56 (70%)

ASA PS, American Society of Anesthesiology Physical Status. Data are expressed as medians (interquartile range) or number of patients (%).

**Table 2:** Univariate and multivariate analyses of variables for OS in patients with pancreatic cancer.

Variables	Univariate Analysis			Multivariate Analysis		
	Odds Ratio	95%CI	*p*	Odds Ratio	95%CI	*p*
Octogenarian	2.69	1.29–5.62	0.008 *	1.19	0.50–2.81	0.698
Female sex	1.02	0.54–1.91	0.950			
BMI <18.5 kg/m^2^	1.18	0.45–3.06	0.740			
Skeletal muscle loss	1.79	0.87–3.68	0.115			
ASA PS ≥3	0.90	0.35–2.31	0.898			
PNI <45	1.14	0.55–2.38	0.723			
mGPS ≥1	2.76	1.11–6.85	0.029 *	2.30	0.85–6.19	0.100
Operation (PD, TP vs. DP)	1.14	0.58–2.24	0.710			
R1 resection	0.97	0.44–2.10	0.929			
Tumor size ≥30 mm	1.73	0.88–3.40	0.114			
Lymph node metastasis	1.62	0.79–3.34	0.189			
Stage ≥IIB	1.85	0.87–3.90	0.108			
Complications (Clavien–Dindo grade ≥III)	0.84	0.38–1.82	0.652			
S-1 adjuvant therapy <6 months	4.62	2.15–9.94	<0.0001 *	3.99	1.76–9.03	0.0009 *

OS, overall survival; CI, confidence interval; BMI, body mass index; ASA PS, American Society of Anesthesiology Physical Status; PNI, prognostic nutritional index; mGPS, modified Glasgow prognostic score. * *p* value < 0.05

**Table 3:** Univariate and multivariate analyses of variables for the discontinuation of S-1 adjuvant therapy.

Variables	Univariate Analysis			Multivariate Analysis		
	Odds Ratio	95%CI	*p*	Odds Ratio	95%CI	*p*
Octogenarian	15.00	1.87–120.39	0.011 *	15.94	1.82–140.06	0.013 *
Female sex	1.30	0.53–3.19	0.566			
BMI <18.5 kg/m^2^	1.27	0.34–4.74	0.723			
Skeletal muscle loss	6.07	2.12–17.42	0.0008 *	6.37	1.99–20.37	0.002 *
ASA PS ≥3	0.98	0.28–3.40	0.975			
PNI <45	1.42	0.49–4.14	0.517			
mGPS ≥1	2.27	0.43–12.11	0.337			
Operation (PD, TP vs. DP)	1.35	0.52–3.47	0.537			
Complications (Clavien–Dindo grade ≥III)	0.72	0.26–2.03	0.536			

CI, confidence interval; BMI, body mass index; ASA PS, American Society of Anesthesiology Physical Status; PNI, prognostic nutritional index; mGPS, modified Glasgow prognostic score. * *p* value < 0.05.

## Data Availability

Data sharing is not applicable to this article. The authors have presented all the necessary information in the manuscript.
